# *Omoglymmius* (*s. str.*) *wukong* sp. n., a new species from Xizang, China (Coleoptera, Rhysodidae, Omoglymmiini)

**DOI:** 10.3897/zookeys.706.14655

**Published:** 2017-10-04

**Authors:** Cheng-Bin Wang, Jan Růžička, Bin Liu

**Affiliations:** 1 Department of Ecology, Faculty of Environmental Sciences, Czech University of Life Sciences Prague, Kamýcká 129, CZ-165 21 Praha 6, Czech Republic; 2 Bin Insect Taxonomy Studio, No.16, Xizhaosi Street, Dongcheng District, Beijing 100061, P. R. China

**Keywords:** China, new species, Omoglymmiini, *Omoglymmius*, Rhysodidae, taxonomy

## Abstract

*Omoglymmius* (*s. str.*) *wukong* **sp. n.** (Coleoptera: Rhysodidae: Omoglymmiini) is described from Xizang, China. Relevant morphological characters of the new species are illustrated with colour plates, and known distribution of the subgenus Omoglymmius in the Himalayan region is mapped.

## Introduction

*Omoglymmius* Ganglbauer, 1891 is the most speciose genus of Rhysodidae (Coleoptera), almost cosmopolitan, but absent from Madagascar, New Zealand, and South America. Bell and Bell (1982) excellently revised *Omoglymmius* and established eleven subgenera to classify the congeneric species. The nominotypical subgenus is the largest with 97 species (Lorenz 2005, Bell and Bell 2009, Hovorka 2015). However, in the fauna of East Asia, only two species in the subgenus Omoglymmius had been recorded before this study, namely *O.* (*s. str.*) *sakuraii* (Nakane, 1973) (China (Taiwan), Japan, Vietnam) and *O.* (*s. str.*) *laticeps* Bell, 1977 (Bhutan, India). In this paper, a new species, *O.* (*s. str.*) *wukong* sp. n., is described and illustrated from Xizang Autonomous Region, China. The new species is compared to the two related species, with some selected and important morphological characters presented in a table.

## Materials and methods

Specimens were relaxed and softened in a hot saturated solution of potassium hydroxide for 4 minutes (for mounted dry specimens) or 8 minutes (for alcohol-preserved specimens), and then transferred to distilled water to rinse the residual potassium hydroxide off and stop any further bleaching. The softened specimens were placed in glycerine and dissected to observe morphological details. After examination, the body parts were mounted on a glass slide with Euparal Mounting Medium for future studies. Habitus photographs were taken using a Canon macro photo lens MP-E 65mm on a Canon 550D. Observations, photographs, and measurements of morphological details were performed using a Zeiss Axio Zoom.V16 motorized stereo zoom microscope with a Zeiss AxioCam MRc 5. Photographs in Figure 6 were taken with an Olympus BX53 microscope with an Olympus DP73 camera. The final deep focus images were created with Zerene Stacker 1.04 stacking software. Adobe Photoshop CS6 was used for final processing. Precise label data are cited, while authors’ remarks and addenda are placed in square brackets; separate label lines are indicated by a slash (/), and separate labels are indicated by a double slash (//). Measurements are averages taken from five specimens. The morphological terminology follows Bell and Bell (1978, 1982). Rhysodid beetles are treated as an independent family, following the publications of Bell (2003), Bousquet (2012), and Makarov (2008).

The material examined for this study is deposited in the following collections and museums (with names of curators in parentheses):

**BITS** Bin Insect Taxonomy Studio, Beijing, China (B. Liu)

**COHP** Collection of Oldřich Hovorka, Prague, Czech Republic

**NHMB** Naturhistorisches Museum, Basel, Switzerland (M. Borer)

**NMEG** Naturkundemuseum, Erfurt, Germany (M. Hartmann)

**NMPC** Národní museum, Prague, Czech Republic (M. Fikáček, J. Hájek)

Measurement criteria in millimetres (mm) are used as follows:

**Antennal length** length between the antennal base and the apex.

**Body length** length between the mandibular apex (mandibles closed) and the elytral apex.

**Elytral length** length between the basal border of elytra and the apex along suture.

**Elytral width** widest part of both elytra combined.

**Eye length** length of a single compound eye in lateral view.

**Eye width** width of a single compound eye in lateral view.

**Head length** length between the anterior apex of clypeus and the posterior margin of temporal lobe along the midline.

**Head width** widest part of head (including compound eyes).

**Pronotal length** length of the pronotum along the midline.

**Pronotal width** widest part of pronotum.

## Results

### Genus *Omoglymmius* Ganglbauer, 1891

Vernacular name: 雕条脊甲属

#### Subgenus Omoglymmius s. str.

Vernacular name: 雕条脊甲指名亚属

##### Omoglymmius (s. str.) wukong sp. n.

Taxon classificationAnimaliaColeopteraRhysodidae

http://zoobank.org/22673184-1DD7-4237-8BDF-C4C270BC6985

Vernacular name: 悟空雕条脊甲

Figs 2 , 3 , 4 , 5 , 6D–F

###### Material examined.

**Holotype**: ♂, CHINA: Xizang, / Chayu County, / Shangchayu Town [上察隅镇], / 16.VIII.2015, / Lu Qiu leg. (NMPC). **Paratypes**: 6♂♂2♀♀, same data as holotype (2♂♂ in BITS, 2♂♂1♀ in COHP, 1♂ in NHMB, 1♂ in NMEG and 1♀ in NMPC); 3♂♂1♀, same data as holotype except: 2000 m, fallen wood, / 24.VIII.2005 (BITS); 1♀, same data as holotype except: 2000 m, *Populus* stump, / 24.VIII.2005 (BITS).

###### Diagnosis.

Head with orbital groove extended before or near the middle of eye, following 1–2 separate coarse dorsal punctures far away from posterior margin of temporal lobe (Figs 2A, C; red arrow in 3A). Pronotal sides gently curved (Figs 2A, C; 3E); (pronotal length)/(pronotal width) = 1.1–1.2 (Figs 2A, C; 3E); outer carina with a distinct oblique microgroove at about basal 1/4 of medial margin (Figs 2A, C; 3E); inner carina impunctate, gradually narrowed in apical part, and weakly undulated at medial margin (Figs 2A, C; 3E); median groove much narrowed in middle part (Figs 2A, C; 3E); marginal groove narrower (Figs 2A, C; 3E); propleuron smooth, almost impunctate except sporadic coarse punctures near margins (Fig. 2B, D); prosternum with sparse coarse punctures and distinct precoxal carinae (Fig. 2B, D). Elytra with stria punctures relatively small (Figs 2A, C; 3F); stria IV with one seta at about basal 2/9, one seta at about apical 2/7 of its length and one seta subapically (Fig. 2A, C). Metasternum with only a few coarse punctures sparsely located along the midline; more coarse punctures closely arranged almost into a row near lateral margins; remainder of disc smooth; a shallow median pit present posteriorly (Figs 2B, D; 3G). Aedeagus with right paramere simply curved at outer margin and expanded in apical part (Fig. 6E). Female profemur without tooth on ventral side (Fig. 2D).

**Figure 1.**
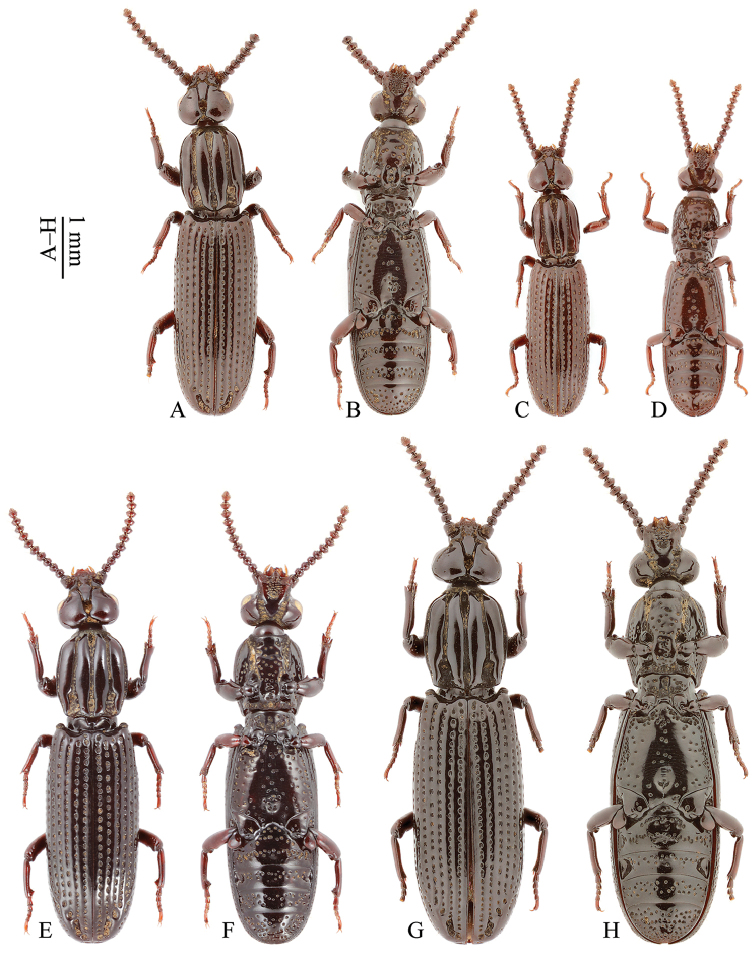
Habitus of *Omoglymmius* (*s. str.*) spp. from East Asia. **A–D** *O.* (*s. str.*) *sakuraii* (Nakane, 1973) (Vietnam: Tam Dao **A–B** ♂ **C–D** ♀) **E–H** *O.* (*s. str.*) *laticeps* Bell, 1977 (**E–F** Bhutan: Thimphu ♂ **G–H** holotype ♀). (**A, C, E, G** dorsal view **B, D, F, H** ventral view).

**Figure 2.**
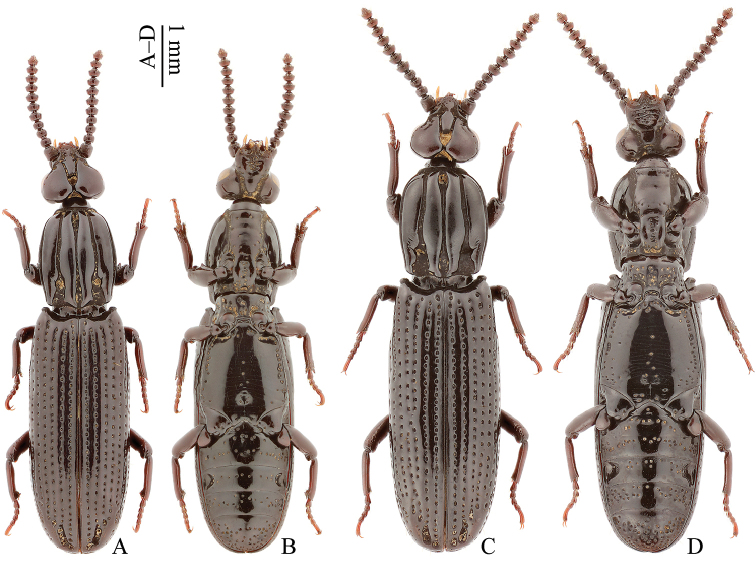
Habitus of *Omoglymmius* (*s. str.*) *wukong* sp. n. (**A–B** holotype ♂ **C–D** paratype ♀). (**A, C** dorsal view **B, D** ventral view).

###### Description.

***Male*.** Medium size, body 6.5–7.0 mm long (6.7 mm in holotype). Length (mm) of different body parts: head (1.0–1.1), pronotum (1.5–1.7), antenna (1.7–1.8), elytra (3.8–4.1); width (mm): head (0.9–1.0), pronotum (1.2–1.3), elytra (1.5–1.6).

*Habitus* (Fig. 2A–B) elongate, rather narrow, lustrous. Body colour mostly blackish brown to black; antennae and legs somewhat reddish brown; mouthparts reddish brown to yellowish brown.

**Figure 3.**
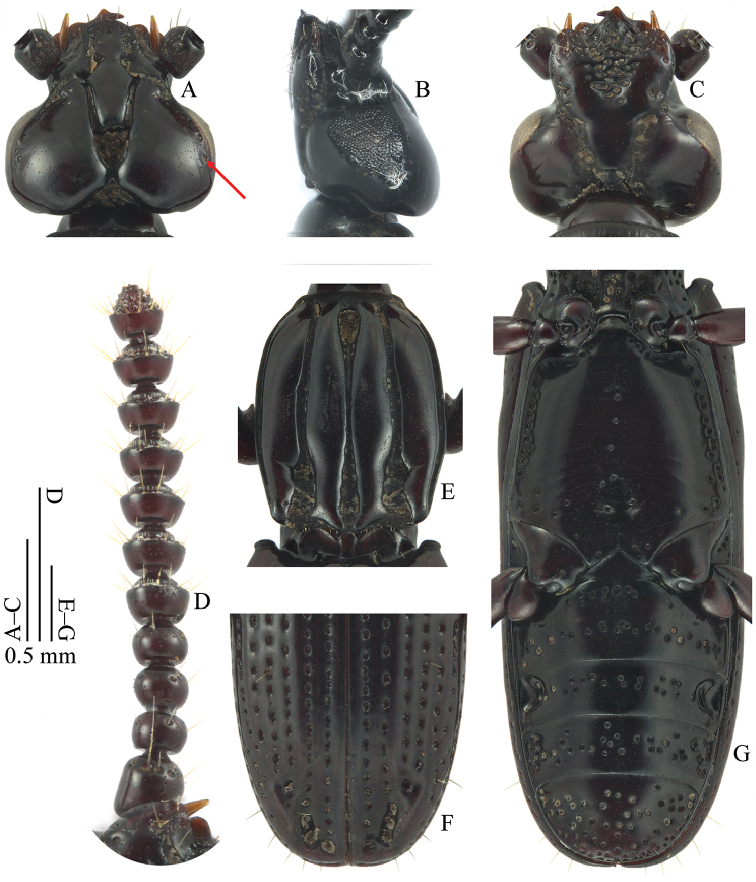
*Omoglymmius* (*s. str.*) *wukong* sp. n. (paratype, ♂). **A–C** head **D** antenna **E** pronotum **F**, elytral apex **G** metasternum & abdomen. (**A, E, F** dorsal view **B** left lateral view **C, D, G** ventral view).

*Head* (Fig. 3A–C) broad, as wide as long. Median lobe short, broad, subtruncate at tip. Frontal space short, nearly V-shaped, margins only shallowly sinuate. Temporal lobes longer than wide; medial angles rounded, contiguous; posteriomedial margin evenly rounded into posteriolateral margin; occipital angle scarcely evident; orbital groove impressed, extended before or near the middle of eye, following one or two separate coarse dorsal punctures far away from posterior margin of temporal lobe (red arrow in Fig. 3A); remainder of temporal lobe smooth except micropunctures; temporal setae absent; postorbital tubercle minute, not pilose, appearing as a slight convexity in lateral view. Eye entire, curvilinearly triangular, length/width = 1.1. Mentum surface coarsely and continuously punctate, with many setae. Antenna (Fig. 3D) without stylet; antennomeres V–X with minor setae in form of subapical rings; basal setae absent; all antennomeres impunctate.

*Pronotum* (Fig. 3E) subelliptical, distinctly narrowed anteriorly and posteriorly, widest at about basal 4/9, length/width = 1.1–1.2. Sides gently curved, hardly sinuate before hind angle; hind angles broadly rounded. Carinae subequal at middle; outer carina with base distinctly narrowed, with medial margin sinuate before base and with a distinct oblique microgroove at about basal 1/4 of its length; inner carina distinctly narrowed in basal part, gradually narrowed in apical part, and weakly undulated at medial margin; both pairs of carinae impunctate except micropunctures. Median and paramedian grooves narrow; median groove much narrower in middle part, opening both anteriorly and posteriorly. Pronotal setae absent. Pronotal hypomeron with many small punctures. Propleuron smooth, almost impunctate except sporadic coarse punctures near margins. Prosternum with sparse coarse punctures; precoxal carinae distinct, sinuate.

*Elytra* (Figs 2A; 3F) elongate, narrow, length/width = 2.2–2.3. Striae impressed, coarsely punctate, punctures relatively small and deep; intervals only slightly convex; stria IV with one seta at about basal 2/9, one seta at about apical 2/7 of its length and one seta subapically; subapical striole with one seta; stria VII with four setae near apex (some specimens with one seta behind the insertion level of hind leg). Metathoracic wings fully developed.

**Figure 4.**
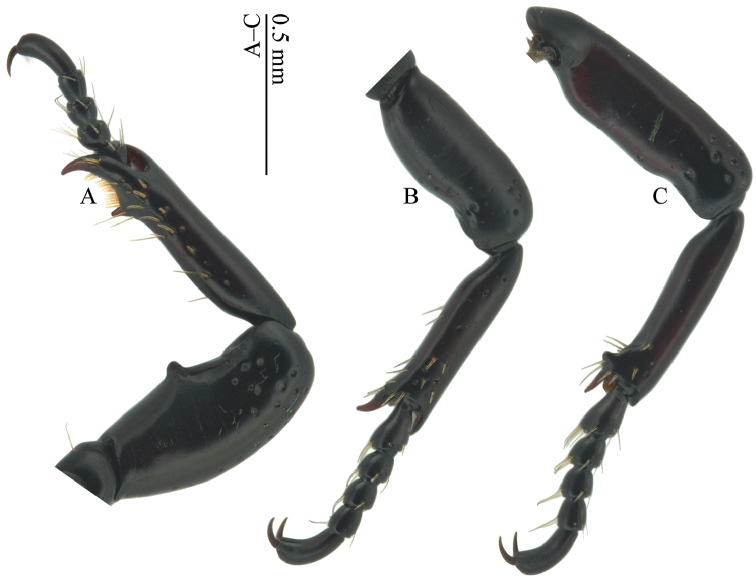
*Omoglymmius* (*s. str.*) *wukong* sp. n. (paratype, ♂). **A** fore leg **B** middle leg **C** hind leg. (**A–C** ventral view).

*Protibia* (Fig. 4A) nearly cylindrical, not swollen at middle; profemur with a large and somewhat rounded tooth at medial position of ventral side. Mesotibia (Fig. 4A) with one curved spur and one minute calcar. Metatibia (Fig. 4C) with one straight spur and one calcar small, subtriangular, obtusely rounded at apex.

Ventral surfaces of *pterothorax* and *abdomen* (Figs 2A; 3G) obviously much smoother than in the related *Omoglymmius* (*s. str.*) *sakuraii* and *O.* (*s. str.*) *laticeps*. Metasternum with only a few coarse punctures sparsely located along the midline; more coarse punctures closely arranged almost into a row near lateral margins; remainder of disc smooth; a shallow median pit present posteriorly. Each abdominal sternum with coarse punctures arranged into two or three irregular transverse rows; sternum IV with deep, round lateral pits; sternum V without visible pits; sternum VI with two setae near apical margin.

**Figure 5.**
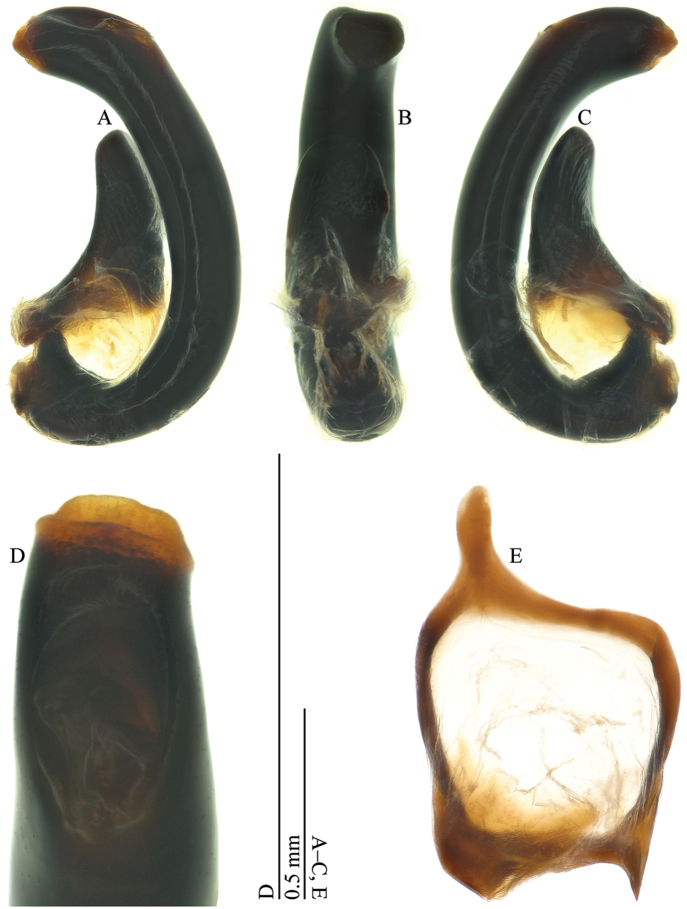
*Omoglymmius* (*s. str.*) *wukong* sp. n. (paratype, ♂). **A–C** aedeagus **D** median lobe **E** genital ring. (**A** right lateral view **B, E** ventral view **C** left lateral view **D** dorsoapical view).

*Genital ring* (Fig. 5E) subquadrate, with long handle, nearly parallel-sided, and rounded at tip.

**Figure 6.**
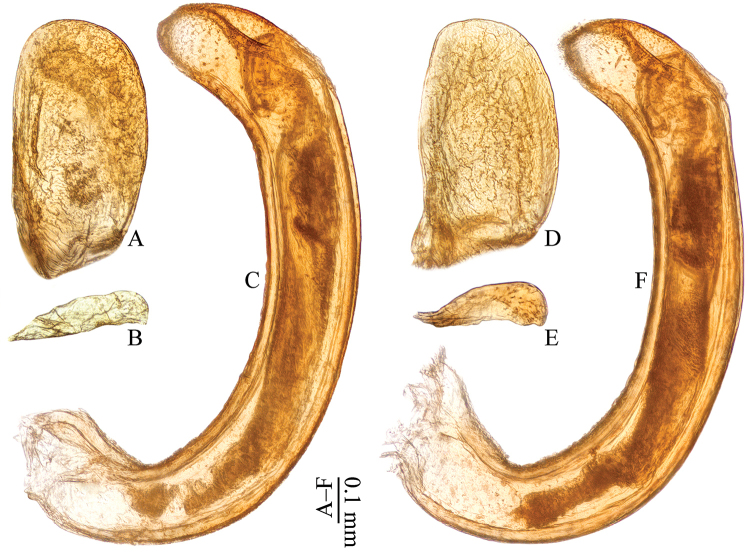
Aedeagi of *Omoglymmius* (*s. str.*) spp. **A–C** *Omoglymmius* (*s. str.*) *laticeps* Bell, 1977 (Bhutan: Thimphu) **D–F** *Omoglymmius* (*s. str.*) *wukong* sp. n. (paratype, ♂) **A, D** left parameres **B, E** right parameres **C, F** median lobes. (**A, D** dorsal view **B, C, E, F** right lateral view).

*Aedeagus* (Fig. 5A–C) with median lobe thick, tubular; opening of apical orifice (Fig. 5D) large, subelliptical; left paramere (Fig. 6D) broad, subelliptical; right paramere (Fig. 6E) small, simply curved at outer margin, expanded in apical part. Endophallus as shown in Fig. 6F.

*Female*. Similar to male in general appearance, but distinguished by the following characteristics (Fig. 2C–D): mentum surface with fewer setae, less coarsely punctate; profemur without tooth on ventral side; meso- and metatibiae without calcars; abdominal sternum IV with lateral pits distinctly larger.

**Etymology.** The specific epithet is from the name of “Sun Wukong”, also known as the Monkey King, a mythological figure who features in a body of legends, which can be traced back to the period of the Song dynasty.

**Figure 7.**
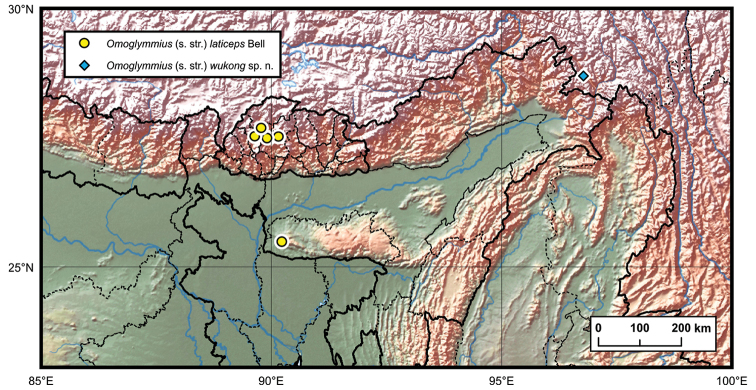
Distribution of *Omoglymmius* (*s. str.*) species from the Himalayan region.

**Distribution.** China (Xizang) (Fig. 7).

**Remarks.** This new species is probably closely allied to the two known species of the subgenus Omoglymmius from East Asia, *O.* (*s. str.*) *sakuraii* (Nakane) and *O.* (*s. str.*) *laticeps* Bell. They resemble each other in general appearance, but detailed comparison of selected morphological characters of importance show their differences (Table 1).

**Table 1.** Selected important morphological differences of *Omoglymmius* (*s. str.*) species from East Asia.

		*O. wukong* sp. n.	*O. sakuraii* (Nakane)	*O. laticeps* Bell
**Body length (mm)**		6.5–7.0	2.8–5.2	6.1–7.1
**Head**	**Orbital groove**	extended before or near the middle of eye, following 1–2 separate coarse dorsal punctures far away from posterior margin of temporal lobe (Figs 2A, C; red arrow in 3A)	extended before the middle of eye, following 2–4 separate coarse dorsal punctures far away from posterior margin of temporal lobe (Fig. 1A, C)	extended after the middle of eye, following 2–4 separate coarse dorsal punctures near posterior margin of temporal lobe (Fig. 1E, G)
**Prothorax**	**Pronotal sides**	gently curved (Figs 2A, C; 3E)	more parallel-sided (Fig. 1A, C)	gently curved (Fig. 1E, G)
	**(Pronotal length)/(pronotal width)**	1.1–1.2 (Figs 2A, C; 3E)	1.3 (Fig. 1A, C)	1.2 (Fig. 1E, G)
	**Outer carina**	with a distinct oblique microgroove at about basal 1/4 of medial margin (Figs 2A, C; 3E)	without microgroove (Fig. 1A, C)	without microgroove (Fig. 1E, G)
	**Inner carina**	impunctate, gradually narrowed in apical part, and weakly undulated at medial margin (Figs 2A, C; 3E)	impunctate, gradually narrowed in apical part, and strongly undulated at medial margin (Fig. 1A, C)	with 2–3 punctures near base, abruptly narrowed in apical part, and weakly undulated at medial margin (Fig. 1E, G)
	**Median groove**	much narrowed in middle part (Figs 2A, C; 3E)	narrow in middle part (Fig. 1A, C)	wide in middle part (Fig. 1E, G)
	**Marginal groove**	narrower (Figs 2A, C; 3E)	narrower (Fig. 1A, C)	wider (Fig. 1E, G)
	**Propleuron**	smooth, almost impunctate except sporadic coarse punctures near margins (Fig. 2B, D)	with many coarse punctures on disc (Fig. 1B, D)	with many coarse punctures on disc (Fig. 1F, H)
	**Prosternum**	with sparse coarse punctures (Fig. 2B, D)	with dense coarse punctures (Fig. 1B, D)	with dense coarse punctures (Fig. 1F, H)
**Elytra**	**Strial punctures**	relatively small (Figs 2A, C; 3F)	relatively large (Fig. 1A, C)	relatively large (Fig. 1E, G)
	**Stria IV**	with one seta at about basal 2/9, one seta at about apical 2/7 of its length and one seta subapically (Fig. 2A, C)	with one seta at about basal 1/5, one seta at middle of its length and one seta subapically (Fig. 1A, C)	with one seta at about apical 2/7 of its length and one seta subapically (Fig. 1E, G)
**Metasternum**		with only a few coarse punctures sparsely located along the midline; more coarse punctures closely arranged almost into a row near lateral margins; remainder of disc smooth; a shallow median pit present posteriorly (Figs 2B, D; 3G)	with more coarse punctures located along the midline; more coarse punctures closely arranged almost into a row near lateral margins; remainder of disc also with a certain number of coarse punctures; a deep median pit present posteriorly (Fig. 1B, D)	with more coarse punctures located along the midline; more coarse punctures closely arranged almost into a row near lateral margins; remainder of disc also with a certain number of coarse punctures; a deep median pit present posteriorly (Fig. 1F, H)
**Aedeagus**	**right paramere**	simply curved at outer margin and more expanded in apical part (Fig. 6E)	unknown	undulate at outer margin and less expanded in apical part (Fig. 6B)
**Female profemur**		without tooth on ventral side (Fig. 2D)	without tooth on ventral side (Fig. 1C–D)	with a small tooth on ventral side (Fig. 1H)

##### Omoglymmius (s. str.) sakuraii

Taxon classificationAnimaliaColeopteraRhysodidae

(Nakane, 1973)

Vernacular name: 樱井雕条脊甲

Figs 1A–D

Nakane 1973: 5 (Rhysodes (Omoglymmius); type locality: Hatsuno, Amami-Ōshima, Japan); Nakane 1978: 130 (Omoglymmius; redescription); Bell and Bell 1978: 75 (Omoglymmius (*s. str.*); taxonomic combination); Bell and Bell 1982: 207 (Omoglymmius (*sensu stricto*); redescription); Bell and Bell 1987: 685 (Omoglymmius (*s. str.*); distribution; remarks; key); Bell and Bell 2000: 74 (Omoglymmius (*s. str.*); distribution).

###### Material examined.

1♂1♀, N Vietnam, 1985 / Tam dao, 3.–11.6. / 900–1400 m / J. Jelínek lgt. // Omoglymmius / (*s. str.*) / sakuraii (Nakane, 1973) / det. O. Hovorka, 1994 (NMPC).

###### Distribution.

China (Taiwan), Japan, Vietnam.

###### Diagnosis.

See Table 1 under *Omoglymmius* (*s. str.*) *wukong* sp. n. above.

##### Omoglymmius (s. str.) laticeps

Taxon classificationAnimaliaColeopteraRhysodidae

Bell, 1977

Vernacular name: 侧头雕条脊甲

Figs 1E–H , 6A–C

Bell 1977: 157 (Omoglymmius; type locality: BHUTAN: Nobding, 41 kilometers east of Wangdi Phodrang, elevation 2800 meters; NHMB); Bell and Bell 1978: 75 (Omoglymmius (*s. str.*); taxonomic combination); Bell and Bell 1982: 206 (Omoglymmius (*s. str.*); redescription); Bell and Bell 2009: 51 (Omoglymmius (Omoglymmius); description of male; distribution).

###### Material examined.

**Type material. Holotype**: ♀, [BHUTAN:] Nobding 41 km O / Wangdi Ph. [Phodrang] 2800 m // Natl. –Hist. Museum / Basel – Bhutan / Expedition 1972 // Omoglymmius / laticeps / det. R. T. Bell // ♀ // (NHMB).

###### Additional material.

1♂, BHUTAN, W / Thimphu env. / 2500 m NN / 01–18.VII.1988 / leg. C. Holzschuh // Omoglymmius / *s. str.* laticeps (Bell) ♂ / det. R. T. Bell // collection / Naturkunde- / museum Erfurt (NMEG); 1♀, BHUTAN, W, distr. / Thimphu, E Dochu La / Menshunang, 2400 m / NN, 07.VII.1988 / leg. C. Holzschuh // Omoglymmius / *s. str.* laticeps (Bell) ♀ / det. R. T. Bell // collection / Naturkunde- / museum Erfurt (NMEG).

###### Distribution.

Bhutan, India (Fig. 7).

###### Diagnosis.

See Table 1 under *Omoglymmius* (*s. str.*) *wukong* sp. n. above.

## Supplementary Material

XML Treatment for Omoglymmius (s. str.) wukong

XML Treatment for Omoglymmius (s. str.) sakuraii

XML Treatment for Omoglymmius (s. str.) laticeps
